# Cubosome-carrying bacterial cellulose membrane as a versatile drug delivery platform

**DOI:** 10.1016/j.mtbio.2024.101000

**Published:** 2024-02-13

**Authors:** Denise Gradella Villalva, Caio Gomide Otoni, Watson Loh

**Affiliations:** a Institute of Chemistry, University of Campinas (UNICAMP), Campinas, SP, 13083-852, Brazil; b Graduate Program in Materials Science and Engineering (PPGCEM) & Department of Materials Engineering (DEMa), Federal University of São Carlos (UFSCar), São Carlos, SP, 13565-905, Brazil

**Keywords:** Liquid crystalline nanoparticle, Microbial cellulose, Biocellulose, Biomembrane, Immobilization, Drug release

## Abstract

Using advanced nanotechnology membranes has opened up new possibilities in the field of biomedicine, particularly for controlled drug delivery and especially for topical use. Bacterial cellulose membranes (BCM), particularly, have gained prominence owing to their distinctive attributes, including remarkable water retention, safety, biodegradability, and tunable gas exchange. However, they are aqueous matrices and, for this reason, of limited capacity for incorporation of apolar compounds. Cubosomes are lipid nanoparticles composed of a surfactant bicontinuous reverse cubic phase, which, owing to their bicontinuous structure, can incorporate both polar and apolar compounds. Therefore, these particles present a promising avenue for encapsulating and releasing drugs and biomolecules due to their superior entrapment efficiency. In this study, we aim to extend earlier investigations using polymeric hydrogels for cubosome immobilization, now using BCMs, a more resilient biocompatible matrix. Phytantriol cubosome-loaded BCMs were prepared by three distinct protocols: ***ex situ*** incorporation into wet BCMs, ***ex situ*** incorporation by swelling of dry BCMs, and an ***in situ*** process with the growth of BCMs in a sterile medium already containing cubosomes. Our investigation revealed that these methodologies ensured that cubosomes remained integral, uniformly distributed, and thoroughly dispersed within the membrane, as confirmed using Small-Angle X-ray Scattering (SAXS) and high-resolution confocal microscopy. The effective incorporation and sustained release of diclofenac were validated across the different BCMs and compared with hyaluronic acid (HA) hydrogel in our previous studies. Furthermore, the resistance against cubosome leaching from the three BCM and HA hydrogel samples was quantitatively evaluated and contrasted. We hope that the outcomes from this research will pave the way for innovative use of this platform in the incorporation and controlled release of varied active agents, amplifying the already multifaceted applicability of BCMs.

## Highlights

• Bacterial cellulose membrane embedding cubosomes for drug delivery.
• Both in situ and ex situ protocols yield uniformly distributed cubosomes.
• Cubosomes’ bicontinuous structure, incorporation, and controlled-release properties are maintained across all protocols.

## Graphical abstract

**Figure:**
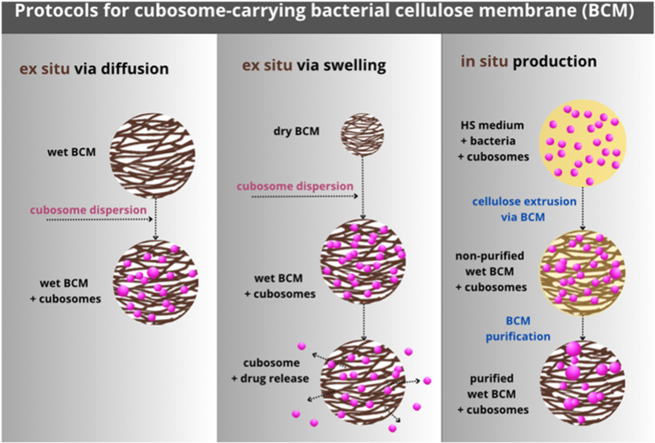

## 1 Introduction

Cellulose stands out as a prominent natural polymer serving as a building block for materials assembly owing to its innate robustness and versatility in multi-scale dimensions, architectural geometry, and bulk and surface functionalities [1]. Cellulose-producing organisms benefit from the structural role played by this biogenic polysaccharide, as observed in plant cell walls and microbial biofilms. In biofilms, cellulose is synthesized by acetic acid bacteria such as *Komagataeibacter xylinus*, following the metabolization of carbon and nitrogen. It is then extruded as a complex and widely entangled hydrated nanofibrillar network, characteristically retaining approximately 99% water [2].

Albeit chemically identical to its plant-derived analogue, bacterial cellulose (BC) boasts a higher purity, devoid of lignin and hemicelluloses. This purity, combined with the significant thermal and mechanical stabilities of the hydrogels it forms, has spurred numerous applications across the biomedical, pharmaceutical, and cosmetic sectors [3]. The use of BC as a temporary skin substitute in the healing of complex burn wounds and chronic venous ulcers is a good example of its versatility [2].

Although boasting suitable features for wound dressing, such as being semipermeable to gases and providing moisture to the damaged skin, BC itself features no antimicrobial activity capable of preventing wound infections. This has been addressed by the chemical or physical immobilization of antimicrobial agents onto BC. While the former ensures a leaching-free approach but is only effective upon direct contact, the latter often requires a further strategy to control the release kinetics of the guest molecules from BC membranes (BCM) [4].

Cubosomes are lipid nanoparticles exhibiting a liquid crystalline structure, characterized by a reverse bicontinuous cubic phase. They excel in the high efficiency of entrapment of hydrophilic, hydrophobic, and amphiphilic guest molecules within the interfacial region bridging their polar and apolar domains [5]. Furthermore, cubosomes can be designed to contain lipid components linked to drugs or proteins to perform specific functions in medical therapies or sensing [[6], [7], [8]].

Glyceryl monooleate and phytantriol are the predominant compounds utilized for cubosome formation, which are typically stabilized as colloidal dispersions using nonionic surfactants. Recent advancements have seen the incorporation of cationic surfactants, such as cetyltrimethylammonium bromide (CTAB) and dodecyltrimethylammonium bromide (DTAB), yielding positively charged cubosomes [9]. These dispersions, as well as their parent bulk phase, have been used in various applications in the biomedical field, particularly for drug release, *e.g.* for cancer treatment [10,11]. In an earlier study, our team characterized cubosomes immobilized within crosslinked hyaluronic acid hydrogels. We determined that while they present a viable platform for drug delivery, their effectiveness is influenced by the degradation of the hydrogel [9].

In this work, we expand upon our previous research with a systematic investigation into the immobilization of phytantriol cubosomes within BCM. This is a more resilient biocompatible matrix that holds promise for prolonged sustained release. Herein, three distinct protocols were employed for cubosome loading, namely: i) ***ex situ* via diffusion**, *i.e.*, a wet BCM membrane, produced in a cubosome-free culture medium, was immersed into a cubosome dispersion; ii) ***ex situ* via swelling**, *i.e.*, a freeze-dried BCM, produced likewise, was reswollen in a cubosome dispersion; and iii) ***in situ production*** by adding growing BC-producing strains in a culture medium consisting of a cubosome dispersion added to the nutrients and followed by the production of the cubosome-loaded BCM.

Cubosomes were assessed regarding stability of their liquid-crystalline structure, size, and spatial distribution patterns within the membrane. Cubosome distribution within the BCM was elucidated using high-resolution confocal microscopy, and their structural integrity was confirmed using small-angle X-ray scattering (SAXS). The effects of cubosome content in the BC-producing medium and its distribution within the BCM were assessed, and cubosome leaching from the BCM was also monitored. Finally, cubosomes were loaded with a model drug, sodium diclofenac (SD), a widely-used nonsteroidal anti-inflammatory drug widely used for osteoarthritic pathologies. SD delivery profiles from cubosome loaded within BCMs were evaluated by tracking the amount released under varying conditions.

## 2 Experimental section

### 2.1 Materials

**Fig. 1:**
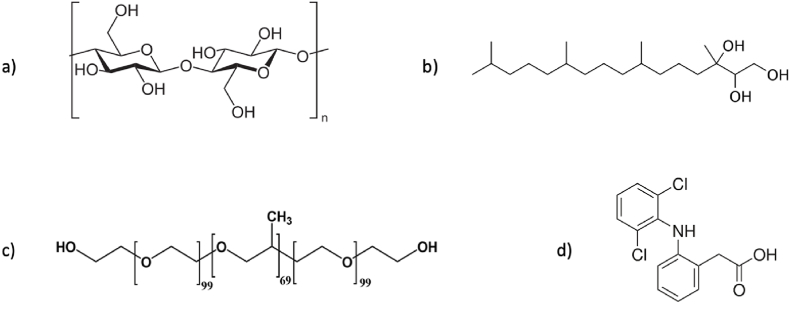
Chemical structures of a) the repeating unit of cellulose, b) phytantriol, c) Pluronic® F-127, and d) diclofenac acid. Cubosomes are based on b, kinetically stabilized by c, loaded with d, and immobilized in a.

Cosmetic-grade phytantriol (3,7,11,15-tetramethyl-1,2,3-hexadecanetriol) with a purity of 97% was provided by DSM company and purchased from Alianza Magistral (SP, Brazil). Various reagents and materials were acquired from Merck-Sigma-Aldrich (Brazil), including: Pluronic® F-127, Nile red (NR), phosphate buffered saline (PBS) at 1 mM, sodium diclofenac, methanol, acetonitrile, glucose, meat peptone, yeast extract, anhydrous disodium phosphate (Na_2_HPO4), citric acid monohydrate, semipermeable (cut-off of 12–16 kDa) dialysis membranes, and 0.20-μm syringe filters. Deionized water (Milli-Q, Millipore Corp., Bedford, MA, USA) was employed to prepare all aqueous solutions. The chemical structures pertinent to cubosome formulation, selective dye, model drug, and cellulose are depicted in Fig. 1.

### 2.2 Cubosome preparation

Sterile cubosomes were prepared based on a protocol described elsewhere [12], with further accurate control over contamination. Briefly, 0.2 g of phytantriol and 0.06 g of Pluronic® F-127 were autoclaved at 121 °C and 200 kPa for 20 min inside a glass vial. Subsequently, 5 mL of pre-autoclaved PBS was introduced to this mixture. After cooling to room temperature, the mixture was heated to 40 °C until phytantriol flowed freely, followed by 1 min of vortexing. Subsequently, the aqueous volume was completed to 10 mL with sterile PBS, resulting in 2% (w/v) of phytantriol and 0.6% (w/v) of Pluronic® F-127. Then, the system was tip-ultrasonicated (3-mm tip; 500 W; Ultrasonic Processor VCX series, Sonic & Materials Inc., Newtown, CT, USA) operating at 40% amplitude and 15s/3s ON/OFF pulse cycles for 30 min. A milky cubosome dispersion in water was formed. Cubosomes were loaded with the model drug through the same protocol but including 0.02 g of sodium diclofenac (10 wt% to phytantriol) dissolved in PBS and sterilized by passing via 0.20-μm syringe filters, resulting in a 0.2% (w/v) content. All procedures were carried out within a laminar flow chamber after autoclaving.

### 2.3 Bacterial cellulose membranes

The BCMs were grown over the surface of liquid Hestrin-Schramm (HS) medium after culturing the *Komagataeibacter xylinus* strain at 28 °C in solid HS culture medium – 2% (w/v) glucose, 0.5% (w/v) peptone, 0.5% yeast extract, 0.27% (w/v) anhydrous disodium phosphate (Na_2_HPO4), and 0.115% (w/v) citric acid monohydrate – followed by growing in 150 mL of liquid HS medium – 4% (w/v) glucose, 0.4% yeast extract, 0.073% (w/v) magnesium sulfate, and 0.2% (w/v) potassium phosphate, in static condition – at 28 °C for 3 d. The liquid medium had been first sterilized at 121 °C and 200 kPa in an autoclave for 20 min. Slow and rotative movements promoted the mixing of bacterial cells with the culture medium under the biofilm, then 100 mL of the medium was poured onto a 32 cm × 52 cm plastic tray containing 700 mL of HS medium. BCM was allowed to grow in a bacteriological incubator at 28 °C for 6 d before the wet membranes were purified (section 2.5., BCM purification). The BCM images are shown in Fig. 5.

### 2.4 Cubosome immobilization into BCM

Three distinct protocols have been evaluated and compared, namely: (a) ***ex situ via diffusion***, *i.e.*, immersing the wet BCM, produced in a cubosome-free culture medium, in a cubosome dispersion; (b) ***ex situ via swelling***, *i.e.*, producing BCM in a cubosome-free culture medium, freeze-drying the wet BCM, and reswelling it in a cubosome dispersion; and (c) ***in situ production***, *i.e.*, adding growing BC-producing strains in a culture medium consisting of a cubosome dispersion added to the nutrients.

For protocol (a) ***ex situ* via diffusion**, wet BCM (*ca.* 1 cm^2^) was immersed into 5 mL of cubosome dispersion for 48 h. For protocol (b) ***ex situ* via swelling**, the BCM was freeze-dried and *ca.* 0.1 g of dry BCM was allowed to reswell by immersion for 48 h in 1 mL of cubosome dispersion. In both cases, cubosome dispersions were prepared in PBS at 2% (w/v) of phytantriol. Finally, for protocol (c) ***in situ production***, the preformed cubosome dispersion was combined with the culture medium for cubosomes to be immobilized during bacterial growth and BCM production. The volume ratio between growing BC-producing strains (100 mL) and liquid HS medium (700 mL) was fixed at 14.3%. Two cubosome dispersion/liquid HS medium ratios (10 and 30 vol%) were tested.

### 2.5 BCM purification

The cellulose produced by Gram-negative bacteria generally promotes various inflammatory responses due to the lipopolysaccharide present in the bacteria membrane, but BC can be extracted in pure form, free of lipopolysaccharides using NaOH in the purification step [13]. The BCMs, loaded or not with cubosomes, were purified in three steps, as follows: (i) washing in 1.75 L of deionized water in an ultrasound bath for 6 min; (ii) heating at 55 °C for 30 min in 1.75 L of alkaline medium (0.1 M NaOH); and (iii) equilibrating at neutral pH in 1.75 mL of deionized water for 15 min [14]. After, the BCM was used as produced for the ***ex situ via diffusion*** and ***in situ production*** protocols and at freeze-dried condition for the ***ex situ via swelling*** protocol.

### 2.6 Small angle X-ray scattering (SAXS) measurements

Experiments were conducted utilizing a Xenocs XEUSS™️ SAXS equipment housed at the Institute of Physics of the University of São Paulo. X-ray radiation, generated by a GENIX™️ source (Cu Kα, λ = 1.54 Å) and featuring a 0.7 mm × 0.7 mm spot size, sufficiently broad to randomly sample multiple nanoparticles, was employed. The cubosome-carrying BCMs were positioned within a 0.5-mm-thick metal support, sandwiched by two Kapton sheets, and measured in air at a controlled temperature of 25 °C. Data were captured via a Dectris Pilatus™️ 300k detector operating with a sample-to-detector distance of 0.9 m. The scattering angle (2θ) varied between 0.12° and 4.59°, producing *q* values ranging from 0.09 to 3.27 nm^−1^. Results were compiled from four frames (of 30 s each) per sample. Cell parameters (a) for the liquid crystalline dispersions were determined based on relative peak positions in the SAXS curves (q), relative to the Miller indices (hkl), employing Eq. (1):

Eq. (1) $${\left(\frac{q}{2\pi }\right)}^{2}={\left(\frac{1}{a}\right)}^{2}\left(h^{2}+k^{2}+l^{2}\right)$$

### 2.7 High-resolution confocal microscopy

Images of fluorescent cubosomes (labeled with Nile red) immobilized into BCM were obtained by high-resolution confocal microscopy on a Zeiss LSM880 Airyscan (Carl Zeiss AG, Germany) microscope, with 488 nm excitation and emission detected in the 550–750 nm range with a smart gain set of 844. Images were acquired at 25 °C by using an EC Plan-Neofluar 10x/0.30 Dry (WD: 5.2 mm) lens (4x zoom) and image dimensions of 170 μm × 170 μm (1024 px x 1024 px) as well as using a C Plan-Apochromat 63x/1.4 Oil DIC (WD:0.14 mm) lens (2x zoom) and images of dimension 27 μm × 27 μm (1024 px x 1024 px). The images were analyzed using the ImageJ software. At least three images of independent samples were chosen to represent each system or condition best.

### 2.8 Swelling capacity

The swelling ratio (SR) was determined by swelling freeze-dried BCM specimens of known dry weight (*ca.* 0.4 mg) and dimensions (*ca.* 2 mm × 0.8 cm x 0.8 cm) in deionized water, PBS, and cubosome dispersions, separately, for 72 h at 25 °C. The swollen membranes were weighed after removing excess liquid with a filter paper. The SR (%) was calculated using Eq. (2), where *W* represents the mass, either swollen (*W*_*S*_) or dry (*W*_*D*_). The experiments were carried out in at least three repetitions, and the measurements were run in triplicates. The results are presented as mean ± standard deviation values.

Eq. (2) $$SR\left(\%\right)=\frac{\left(W_{S}-W_{D}\right)}{W_{D}}*100$$

### 2.9 Scanning electron microscopy (FE-SEM)

The morphologies of freeze-dried BCMs (coated with Au/Pd alloy and Ir metal sputtering on a MED020 – Baltec) were analyzed on a Quanta-FEG 250 (FEI) field-emission scanning electron microscope (SEM) by secondary electrons (Everhart-Thornley detector – ETD), operating at 2.0 kV.

### 2.10 Cubosome leaching from BCM

The leaching profiles of cubosomes immobilized in BCM were followed for one week. Freeze-dried BC (0.1 g) added with 1.0 mL of cubosome dispersion was immersed in 10 mL of PBS into a Falcon tube. Aliquots of 0.5 mL were withdrawn after 1, 3, 9, 24, 48, 72, 96, and 168 h. PBS was added to replace each withdrawn aliquot. Methanol (0.5 mL) was added to each aliquot and vortexed to disrupt the cubosomes. After this mixture, the aliquots were again diluted 400 times in PBS/methanol (1:1). Then, 5 μL were injected and quantified using ultra-performance liquid chromatography-mass spectrometry (UPLC-MS) [15]. The UPLC instrument comprised the Acquity model from Waters, the BEH C18 1.7 μm (2.1 × 50 mm) column, and a gradient elution profile of 0.3 mL min^−1^ (solvent A: water + 0.1% (w/v) formic acid; solvent B: acetonitrile + 0.1% (w/v) formic acid) and a gradient program of 95:5 (1 min), 5:95 (8–9 min), and 95:5 (11 min) as the eluent (total run time of 12 min). The MS measurements were carried out on a Quattro Micro API from Waters, using SIR (capillarity of 3.3 kV, cone of 16 V, extractor of 3 V, RF lens of 0.2 V, source temperature of 120 °C, and desolvation temperature of 350 °C) with an electrospray interface in the negative mode for selective ion monitoring for *m*/*z* 375 and 391. All experiments were repeated at least three times, and the measurements were run in triplicates. The results are presented as mean ± standard deviation values.

### 2.11 Encapsulation efficiency

The fraction of SD (model drug) that was not loaded within 1 mL of cubosomes was separated through ultrafiltration using Amicon Ultra filters (Amicon Ultra-4, 10,000 MWCO, Millipore), and the mixture was centrifuged at 5000 rpm for 30 min at 25 °C. Subsequent filtrates were diluted using a PBS/methanol mixture (1:1), and their content was analyzed via spectrophotometry at 276 nm. The encapsulation efficiency (EE; %) was calculated using Eq. (3), wherein *W*_*T*_ represents the total drug mass loaded within the cubosome formulation and *W*_*F*_ is the amount of free drug in the filtrate determined by spectrophotometry. All experiments were repeated at least thrice and measurements were run in triplicates. The efficiency of this method was previously demonstrated [9] when quantifying the phytantriol using ultra-performance liquid chromatography-mass spectrometry (UPLC-MS) [15], confirming the absence of phytantriol in the filtrate.

Eq. (3) $$EE\left(\%\right)=\frac{W_{T}-W_{F}}{W_{T}}*100$$

### 2.12 Drug delivery profiles

The release profile of SD from cubosomes was determined in terms of its accumulated mass (%) to the initial SD content in the BCM. The samples were enclosed in dialysis bags and immersed in 10 mL PBS. Aliquots of 500 μL were collected at 1, 3, 6, 9, 12, 24, 48, 72, 96, 120, 144, and 168 h, and 500 μL of PBS were added back to replace each withdrawn aliquot. SD content was quantified using HPLC on a Prominence (series LC-20A; Shimadzu) chromatograph using a Symmetry C18 column and acetonitrile as eluent. An area (Y) *versus* mass (X) calibration curve (Y = 2e^9^X; R^2^ = 0.99) was determined from the SD standard at 0.18 mg mL^−1^ (ranging from 0.001 g to 0.006 g) prepared in PBS. Each experiment was carried out at least three times. Results are expressed as mean ± standard deviation values.

To determine the mechanism of drug release, the semi-empirical Korsmeyer-Peppas model was fitted to the release profiles. The model is given by Ref. [16]:

Eq. (4) $$\text{Mt}/M\infty =k_{\text{KP}}\cdot t^{n}$$

where Mt/M∞ is the fractional drug release, t is the release time, **k**_KP_ is the Korsmeyer-Peppas release kinetic constant characteristic of the drug/system (particle and matrix), and **n** is an exponent that characterizes the release mechanism [16]. The data were fitted using the OriginPro9 software.

## 3 Results and discussion

### 3.1 Cubosome morphology and structure

The initial phase of this investigation was dedicated to evaluating the distribution of cubosomes within the BCM matrix. By using 30 wt% Pluronic® F-127 (relative to phytantriol) for nanoparticle stabilization, we adhered to a previously established protocol to guarantee kinetically stable dispersions [10]. All resulting formulations exhibited a milky appearance with a monomodal particle size distribution. The mean diameters were around 190–200 nm, based on intensity measurements, and polydispersity indices varied between 0.1 and 0.2, both from Dynamic Light Scattering measurements. Incorporating diclofenac at 10 wt% (relative to phytantriol) slightly decreased the mean cubosome size from 193 ± 2 to 187 ± 2 nm. Given that SD bears a negative charge at pH 7.2, the zeta potential values for SD-loaded cubosome dispersions shifted to more negative values, from −2 to −7 mV, as reported previously (including the graphs) by our group [9]. In our group's previous studies, the internal structures of the reverse cubic phase of cubosomes, both empty and loaded with 0.2% (w/v) SD, have been comprehensively characterized using SAXS [9], pointing to the Pn3m space group.

### 3.2 Cubosome-carrying bacterial cellulose membranes

**Fig. 2:**
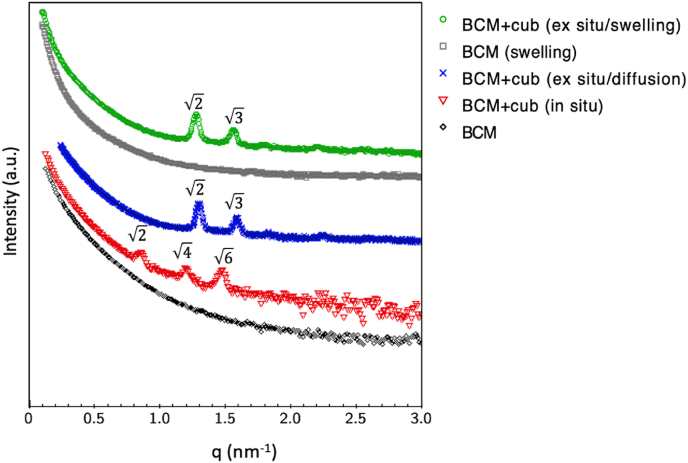
SAXS curves of empty cubosomes immobilized either ***ex situ* by diffusion** (blue crosses), ***ex situ* by reswelling** (green circles) and ***in situ*** (red triangles). The reference curves of bacterial cellulose membrane (BCM) in buffer are represented for original BCM (black) and for lyophilized and swollen BCM (gray). (For interpretation of the references to color in this figure legend, the reader is referred to the Web version of this article.)

Cubosomes within the BCMs were evaluated for their stability, dimensions, distribution patterns, and structural integrity. When immobilized into BCM using ***ex situ*** protocols (both ***via* diffusion** and ***via* swelling**), the cubosomes exhibited SAXS profiles (Fig. 2, blue and green curves) with Bragg peaks at relative positions corresponding to ratios of $\sqrt{2}$ and $\sqrt{3}$. This pattern confirms the reverse cubic bicontinuous internal structure of the nanoparticles, with peaks indexed as (110) and (111), consistent with the Pn3m space group [17] (diamond type). The Pn3m symmetry of empty cubosomes and their characteristic distances remained consistent whether incorporated into BCM ***via* diffusion** or ***via* swelling**. According to the ***in situ*** immobilization protocol, cubosomes were mixed with the HS liquid during the BCM production, leading to immobilization as the matrix grew. However, this method altered the internal structure of cubosomes, as evidenced by Bragg peaks at relative positions with ratios of $\sqrt{2}$, $\sqrt{4}$, and $\sqrt{6}$ (Fig. 2, red curve). These peaks correspond to the (110), (200), and (211) planes, indicative of the Im3m space group. Such transitions between different bicontinuous cubic structures are expected for cubosomes [9] when in the presence of a third component (such as a drug or an ionic lipid) [18] or changes in the dispersion environment (such as increased ionic strength) [5]. Particularly, added salt can screen the charges of the internal components and alter the diameter of the water channels in cubosomes, impacting globally the final structure and promoting phase transitions. The presence of additional salts in the culture medium and the metabolic byproducts of the fermentation process, meaning bacterial lipid residues, likely drove this phase change. Yet, the final characteristics were akin to cubosomes rather than adopting alternative structures.

**Fig. 3:**
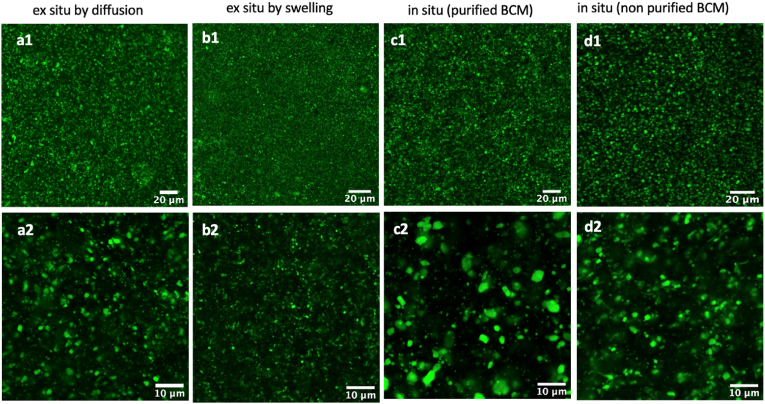
High-resolution confocal microscopy images of cubosomes immobilized in BCMs through different protocols, namely ***ex situ* via diffusion** (a1, a2), ***ex situ* via swelling** (b1, b2) and ***in situ* production** for purified BCM (c1, c2) and ***in situ*** for non-purified BCM (d1, d2) from 10 vol% cubosome in HS medium dispersion. Images in the top row (a1, b1, c1, d1) were acquired using a 10x objective lens (4× zoom, scale bar = 20 μm), while for those in the bottom row (a2, b2, c2, d2), a 63x objective lens (2× zoom, scale bar = 10 μm) was used.

The distribution of fluorescent cubosomes (labeled with Nile red) within BCMs was examined using high-resolution confocal microscopy. Fig. 3 compares images of membranes prepared both via ***in situ*** and ***ex situ*** protocols. Confocal microscopy effectively captures clear images of objects with sizes over approximately 300 nm. However, smaller entities, down to around 140 nm, are also visible, albeit with diminished definition. The images of cubosomes immobilized ***ex situ* via swelling** into BCM were acquired from a BC/phytantriol mass ratio of 5 (0.1 g BCM to 0.02 g phytantriol).

Immersing approximately 1 cm^2^ of wet BCM into a cubosome dispersion (**via diffusion** protocol) yielded a color tonality akin to that observed for both the wet BCM prepared via swelling and the ***in situ*** protocol in which fluorescent cubosomes were mixed with the HS culture. Upon observation with a 10x objective lens, all images (for ***ex situ*** and ***in situ*** protocols) consistently displayed a widespread distribution of cubosomes throughout the BCM, and no sign of extensive aggregation was detected (as seen in Fig. 3 a1, **b1**, **c1**, and **d1**).

Despite the apparent uniform distribution (with 10x objective lens) for cubosomes incorporated ***in situ*** and ***ex situ* via diffusion**, they actually displayed some limited aggregation of cubosomes that led to a few domains of *ca.* 2.5 μm (Fig. 3 a2, d2). The comparison of Fig. 3 c2 and Fig. 3 d2 suggests that the purification step of BCM promotes further aggregation, producing larger domains of *ca.* 5.0 μm in size.

The concentration, size, and surface area of cubosomes and their distribution within the matrix play critical roles in modulating the release kinetics of incorporated compounds and their subsequent transport and destination in the body [16,19].

Nevertheless, the extent of aggregation seems to be rather limited and far from a detrimental coagulation. Therefore, the present results prove that it is possible to grow the BCM in the presence of cubosomes, in an event where this would be required. The signs of agglomeration and changes in the internal structure of cubosomes do not invalidate the use of the ***in situ*** protocol for future drug delivery applications. In specific scenarios, incorporating cubosomes during BCM production might offer benefits, such as one-pot processes and preserving the inherent properties of BCM, which remain unaffected by the freeze-drying step. Nevertheless, further research and optimization of the ***in situ*** protocol remains essential.

Moreover, it is established that the interaction between polymer-coated and lipid-coated nanoparticles exhibits noteworthy variations in bacterial interaction outcomes [20]. In this context, it was observed that bacteria persisted in a viable state and successfully generated the BCM. Using the copolymer to stabilize phytantriol cubosomes is proposed also to prevent nanoparticles from interacting with *K. xylinus* cells.

**Fig. 4:**
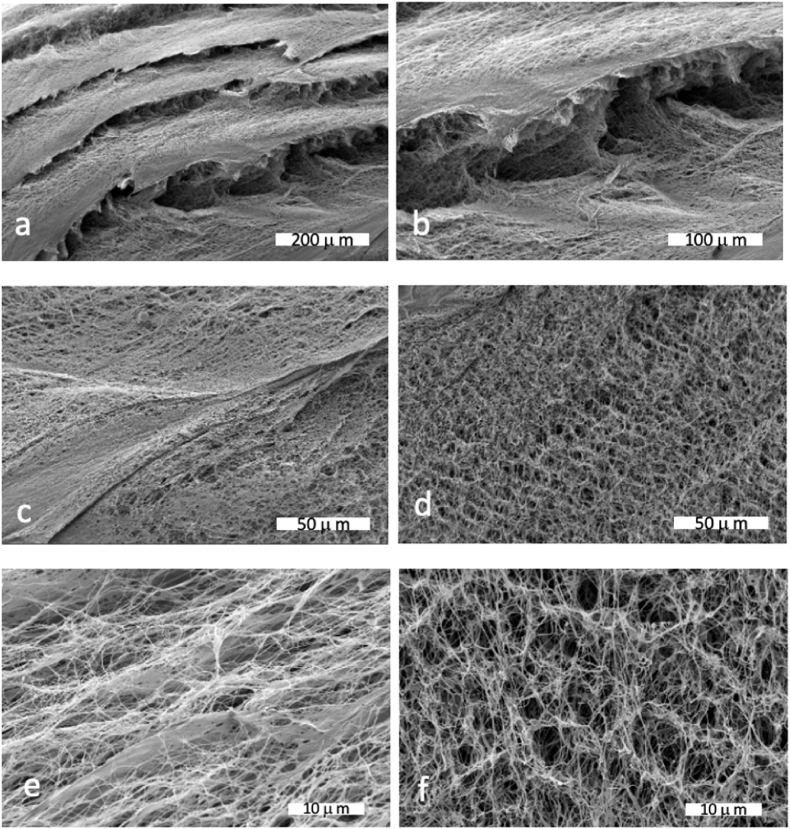
SEM images of flash-frozen and freeze-dried BCMs. The images a) and b) show the stratified structuring of BCM separating internal porous cavities (zoom of 200x and 250x, respectively); c) and d) are magnified to 1000×: c) the denser structures (the interfaces of layers) and d) internally the cavities; e) and f) are images at 4000x magnification and represent the layer and more internal porous structures, respectively.

The morphology of BCMs after freeze-drying was evaluated using SEM (Fig. 4). To minimize the disruption of BCM's porous architecture due to ice crystal formation, samples were flash-frozen using liquid nitrogen before lyophilization. Subsequently, the freeze-dried BCM samples were fractured following a 30-min immersion in liquid nitrogen. SEM images reveal a multilayered membrane structure. Generally, the thickness—defined here as the number of BC layers—is influenced by the duration of BC production. Additionally, the water retention capacity of BCM is modulated by the density of microfibrils and the formation of hydrogen bonds [21,22].

**Fig. 5:**
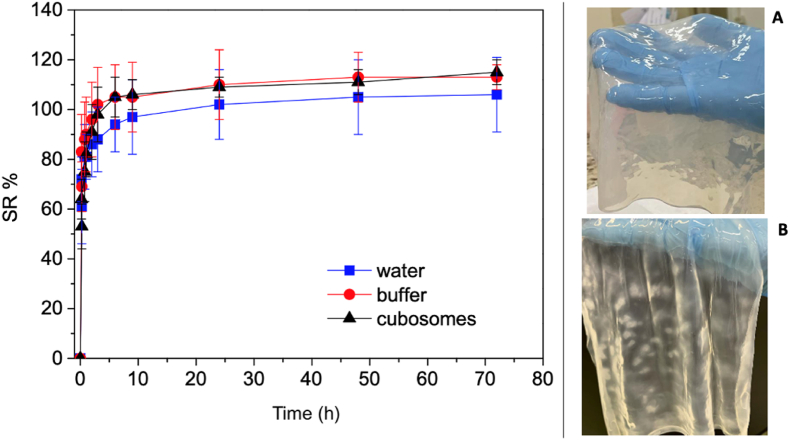
Graphs of swelling ratio (SR) of bacterial cellulose membranes (BCM) immersed in: deionized water (blue squares), in PBS buffer (red circles), and cubosome dispersion in PBS (black triangles). Images of: A) Original BCM for ***ex situ*** by diffusion or to be freeze-dried for ***ex situ*** by swelling and B) BCM prepared incorporating cubosomes by ***in situ*** protocol. (For interpretation of the references to color in this figure legend, the reader is referred to the Web version of this article.)

Our SEM images closely align with existing literature on BCM morphology [23,24]. According to Fig. 4 (a,b), the distance between the layers is lower than 100 μm and in Fig. 4 (c,d,e,f) the more internal porous structures display dimensions smaller than 5 μm. Given these size ranges, cubosomes (separately or clustered) could be entrapped within the mesh of the fibrous network, with their sizes on the order of a few microns.

The evaluation of swelling profiles for freeze-dried BCM serves as a critical parameter in determining the volume fraction of cubosomes that can be effectively immobilized within the matrix, relying on the volume of cubosome dispersion that can be absorbed. Fig. 5 presents the SR of BCM when immersed in deionized water, PBS buffer, and cubosome dispersions in PBS.

Due to the superior dispersion and distribution characteristics observed for cubosomes immobilized into BCM using ***ex situ*** impregnation (**via swelling**), this protocol was chosen for further evaluations of nanoparticle leaching and drug release kinetics in excess of PBS buffer. Furthermore, the ***ex situ* via swelling** protocol allows for precise control over the concentration of cubosomes and the quantity of drug loaded within them.

The BCM required at least 48 h to reach complete swelling, achieving a liquid mass around 110 times its initial dry weight: 105 ± 18 times for deionized water, 113 ± 10 times for PBS, and 111 ± 5 times for cubosome dispersion. Notably, most of the swelling occurred within the initial hours of exposure. According to the literature, variables such as inoculum volume, fermentation duration, and drying techniques significantly influence the BCM's parameters such as thickness, swelling ratio, and drug release kinetics. Indeed, for suppressing pore collapse due to capillary stresses, freeze-drying is by far the most suited method to obtain highly porous BC cryogels featuring higher water retention values and improved properties of interest as far as scaffold [25], in comparison to other methods such as evaporative (xerogels) and oven drying [3].

### 3.3 Cubosome leaching and SD release from BCM using *ex situ* protocol (via swelling)

#### 3.3.1 Cubosome leaching using *ex situ* protocol

**Fig. 6:**
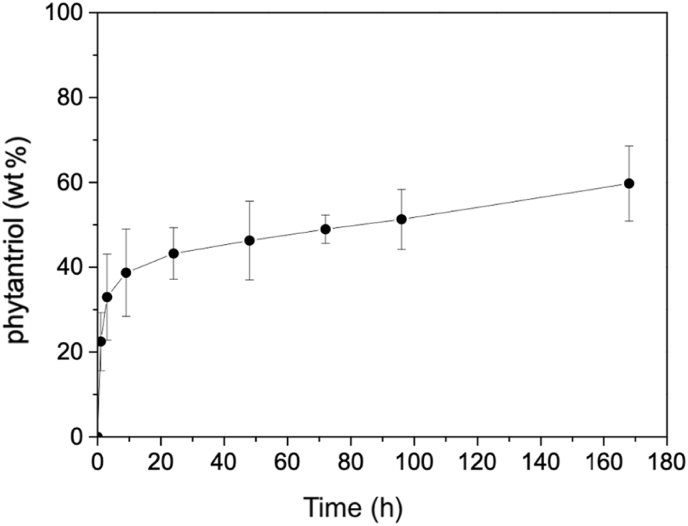
Cubosome leaching from bacterial cellulose membranes as a function of time. The phytantriol content was used to estimate the amount of cubosomes.

Our cubosomes are composed majorly of phytantriol. Therefore, a phytantriol calibration curve by mass spectrometry was used to quantitatively track cubosome leaching from the BCMs [15]. Fig. 6 shows that, during the first 3 h, 33 ± 10 wt% of the initial phytantriol content was released through cubosome leaching. After 72 h, 49 ± 3 wt% of the total phytantriol was released from the membrane. After a week (168 h), cubosomes were still present in BCM because only 60 ± 9 wt% was leached, suggesting some membrane/nanoparticle interactions or the physical entrapment of the latter within the former. After seven days, the membrane remained intact, but the leaching of cubosomes was not monitored thereafter. The structure of the BCM was overall well-preserved in cold water (around 4 °C) for at least six months.

#### 3.3.2 SD release from BCM by *ex situ* protocol

Sodium diclofenac was chosen as a model drug to follow its controlled delivery from the immobilized cubosomes. In a previous study of our group, diclofenac was effectively incorporated in phytantriol cubosomes, resulting in an EE of 96.5 ± 0.1 wt% for a formulation containing 10 wt% SD (with respect to phytantriol) [9]. According to the literature, the 1-octanol/water partition coefficient (K_OW_) of SD is around 4.0–4.5 [26], meaning that diclofenac is rather soluble in both water and octanol (assumed to represent the hydrophobic moiety of biological membranes).

**Fig. 7:**
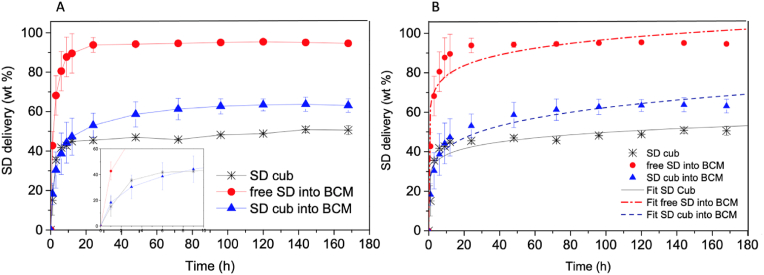
A) Profile of accumulated sodium diclofenac (SD) released from three different systems: SD loaded into cubosome dispersions (black asterisks), free SD loaded directly into bacterial cellulose membranes (BCM) (red circles), immobilized cubosomes (loading SD) into BCM (blue triangles). The inset details the first 10 h of SD delivery. B) Korsmeyer-Peppas model fitting of SD release profiles for: SD loaded into cubosome dispersions (black), free SD loaded into BCM (red), immobilized cubosomes (loading SD) into BCM (blue). (For interpretation of the references to color in this figure legend, the reader is referred to the Web version of this article.)

A comparison of the accumulated amounts released of (i) free SD loaded directly into the BCM, (ii) SD from cubosomes either immobilized into BCM, and (iii) SD from free cubosomes in dispersion (without BCM) is shown in Fig. 7. In general, free SD within BCM was more rapidly delivered than SD loaded into cubosomes and then SD loaded into cubosomes immobilized in BCM. In all cases, the amount of incorporated SD was 10 wt% with respect to phytantriol, and for systems without cubosomes, the same total free drug quantity was directly loaded into BCM.

During the first 12 h, 90 ± 10% of SD was delivered from BCM (that did not contain cubosomes) compared to 45 ± 1% from cubosomes dispersions (without BCM) and 47 ± 9% of SD from cubosomes-carrying BCM. At 72 h, 95 ± 10% of free SD was delivered from BCM compared to 46 ± 1% from cubosomes dispersions and 61 ± 9% of SD from cubosomes immobilized into BCM. After one week, 95 ± 5% of free SD had already been delivered from BCM compared to 51 ± 2% from cubosome dispersions and 63 ± 3% of SD from cubosomes-carrying BCM. The delivery of SD from cubosomes dispersions (without a matrix) has been studied in our previous work, using the same experimental conditions and leading to similar results [9]. It is essential that the same conditions are used, including temperature, pH, cubosome composition, drug type and loaded amount, stirring of the release medium, etc. to enable proper comparison.

According to Fig. 7, the cubosome-carrying BCMs retained greater amounts of SD than the free drug in BCM. Cubosomes show a slower SD release than BCM due to their large surface area and higher capacity to retain SD into the water channel than the porous BCM. After 12 h, there is a divergence in that more SD is released from cubosomes incorporated into BCM, with respect to just cubosome dispersion, probably due to BCM–Cub interaction across the network. More studies are necessary to understand the cubosome destabilization process. These results emphasize that the BCM features and the cubosomes themselves are important factors to control drug release in a synergistic manner.

We quantified the free drug that passed through the cellulose dialysis membrane. In order to attain a deeper perception of the mechanisms governing SD release from the samples, release profiles were fitted to the semi-empirical Korsmeyer-Peppas model (Eq. (4)) [27]. This model is particularly suitable for discerning release mechanisms when one or more types of phenomena take place. The acquired kinetic constant characteristic values (kKP), which are associated with the structural attributes of the drug/carrier/matrix system and the interactions among these components, delineated the following release rate trends for SD: SD into BCM >SD cub >SD cub into BCM, i.e., 63.6 > 29.7 > 27.7.

The optimal adjustments utilizing the Korsmeyer-Peppas model (R^2^_adj_) were observed for the SD cub into BCM systems, resulting in an R^2^ value of 0.953, thereby confirming the suitability of the Korsmeyer-Peppas model (refer to Fig. 7B and Table 1). The concentration gradient over time is elucidated by the value of **n** in Eq. (4). When **n** is lower than 1 (n < 1), it indicates a reducing concentration gradient over time and corresponds to a Fickian diffusion release during which the solvent penetration is the rate-limiting step [16]. To sum up, the aim for the drug release will depend upon the characteristics of the administered drug, the affinity of the drug with the nanoparticle, and the chemical compatibility between nanoparticles and the hosting matrix.

**Table 1:** Parameters of SD drug delivery using Korsmeyer-Peppa model.

SD delivery system	k_KP_	n	R [2] _adj._
SD into BCM	63.6	0.091	0.904
SD cub into BCM	27.7	0.176	0.953
SD cub	29.7	0.112	0.871

## 4 Comparison between SD release from BCM and from hyaluronic acid hydrogels

**Fig. 8:**
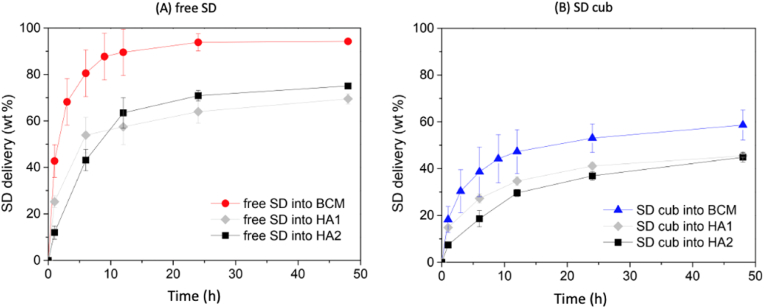
Percentage of sodium diclofenac (SD) released from bacterial cellulose membranes (BCM) and hyaluronic acid (HA) matrices: (A) free SD loaded directly into BCM (red circles), HA1 (gray diamonds), and HA2 (black squares); (B) SD-carrying cubosomes loaded into BCM (blue triangles), into HA1 (gray diamonds), and into HA2 (black squares). (For interpretation of the references to color in this figure legend, the reader is referred to the Web version of this article.)

In a previous work [9], cubosomes immobilized in hyaluronic acid (HA) hydrogels were investigated at different crosslinking degrees with promising outcome for biomedical applications. Here, the accumulated SD release from cubosome-carrying BCM was compared to that of cubosome-carrying HA hydrogel (Fig. 8), both using the ***ex situ* via swelling** protocol, at intermediate (HA1) and high (HA2) crosslinking degrees of hydrogels. It is worth highlighting that PBS at pH 7.2 was used in all experiments, including in our previous contribution, enabling controlling the pH- and ionic strength-dependencies of SD release. Furthermore, under neutral conditions (pH = 7.2), SD-loaded cubosomes exhibited a negative zeta potential value, but unlike HA BCM lacks electric charge. In addition to the physical entrapment, the Pluronic® F-127-stabilized cubosomes are believed to interact primarily through hydrogen bonding with the bacterial cellulose matrix, despite the presence of water.

Both systems show interesting results, with the highly crosslinked HA membrane being more efficient in retaining the cubosomes within its structure, *i.e.*, the impact of cubosome leaching is negligible (under 1%) [9]. Differently, there was significant cubosome leaching from BCMs, as already demonstrated in section 3.3. Yet, BCM is a completely natural material of easy processing that does not require any chemical crosslinking, given the intrinsically entangled nature of the cellulose fibrils, standing out as a robust membrane for topical applications. It is inherently biodegradable by several enzymes found in nature, but not by those found in the human body [28]. Yet, BCM may be advantageous over other hydrogels as its biodegradability could be controlled by buffers, polymers, proteins, and other chemical or physical modifications to enhance its decomposition and bioabsorption in living systems [28]. Cubosome-carrying BCMs could support treatments requiring expressive releases in the beginning with sustained release along the therapy.

As mentioned in section 3.3.1, after a week, cubosomes are still present in BCM and BCM remains intact for months, although the leaching from BCM was followed for only one week. At the same time, cubosome leaching from HA hydrogel is directly associated with the hydrogel degradation rate (related to their crosslinking degree), which does not occur for BCM, this being a clear advantage of BCMs over HA hydrogels. HA hydrogels with an intermediate degree of crosslinking were degraded in around one week, therefore leaching all cubosomes to the solution. Only hydrogels with a high degree of crosslinking remained intact for longer periods, but still, they are less resilient than BCMs.

## 5 Conclusions

Phytantriol-based cubosomes were successfully immobilized into bacterial cellulose membranes using three distinct protocols: two ***ex situ*** and one ***in situ*** approaches. These cubosomes were found to be uniformly dispersed within the BCM across the three protocols. However, some degree of limited agglomeration was observed for both the ***ex situ* via diffusion** and the ***in situ production*** methods, as revealed by high-resolution confocal microscopy images.

Importantly, the cubic bicontinuous internal structure characteristic of cubosomes was preserved in all cases. While immobilizing cubosomes in situ, the presence of other salts, fermentation residues, and/or bacterial lipids in the culture medium likely caused a transition from the typical Pn3m structure of phytantriol-based cubosomes to Im3n, as shown by SAXS.

The complete swelling of the freeze-dried BCM (after 48 h) yielded the incorporation of approximately 99% liquid (water or PBS). Freeze-drying is a suited method to obtain highly porous BCM featuring high water retention.

Leaching profiles revealed that over 50% of the cubosome content remained within the BCM after 72 h, substantiating the potential of this platform for sustained drug delivery. The release kinetics of sodium diclofenac indicated prolonged drug delivery when incorporated within cubosome-carrying BCMs. Importantly, the BCM matrix was resilient for long periods, an advantage over hydrogels previously studied, which disintegrated after some days, leaching cubosomes. In fact, these BCMs could even be reused, if appropriate.

Another important factor related to the application of these delivery systems is the cytotoxicity. We have not assessed this property directly for the present formulations, but their components have been previously investigated. In this respect, phytantriol cubosomes stabilized with F127 were tested over a wide concentration range, encompassing the ones used in the present study, against different cell lines [29,30]. Similarly, several reports have indicated that BCM is non-cytotoxic toward cell cultures, such as fibroblasts [31] and endothelial cells [32].

These findings lend significant support to the feasibility of using BCMs for immobilizing cubosomes in future biomedical applications in drug delivery. This platform offers advantages such as biocompatibility, high moisture retention, cost-effectiveness, resilience, and sustainability, making it a promising vehicle for targeted drug delivery, particularly for dermatological therapies.

## CRediT authorship contribution statement

**Denise Gradella Villalva:** Writing – original draft, Investigation, Data curation. **Caio Gomide Otoni:** Writing – review & editing, Writing – original draft, Data curation, Conceptualization. **Watson Loh:** Writing – review & editing, Supervision, Data curation.

## Declaration of competing interest

The authors declare that they have no known competing financial interests or personal relationships that could have appeared to influence the work reported in this paper.

## Data Availability

Data will be made available on request.
